# Acceptability of the R21/Matrix-M malaria vaccine alongside existing malaria interventions in the trial context

**DOI:** 10.1136/bmjgh-2024-015524

**Published:** 2025-02-03

**Authors:** Halimatou Diawara, Jane Grant, Alassane Dicko, Seydou Traore, Djibrilla Issiaka, Fatoumata Koita, Mehreen Datoo, Mala Sylla, Abdrahmane Boncane Dicko, Issaka Sagara, Daniel Chandramohan, Adrian VS Hill, Brian Greenwood, Jayne Webster

**Affiliations:** 1 Malaria Research and Training Centre, University of Sciences Techniques and Technologies of Bamako, Bamako, Mali; 2 London School of Hygiene & Tropical Medicine, London, UK; 3 Centre for Clinical Vaccinology and Tropical Medicine, The Jenner Institute, University of Oxford and the NIHR Oxford Biomedical Research Centre, Oxford, UK; 4 Ministry of Health and Public Hygiene, Bamako, Mali

**Keywords:** Malaria, Qualitative study, Vaccines, Global Health, Chemoprophylaxis

## Abstract

**Background:**

The R21/Matrix-M malaria vaccine has been shown to provide high protective efficacy against malaria in a phase III trial, and has been recommended for use by WHO. The vaccine will soon be deployed at scale in sub-Saharan Africa. This study aimed to understand the caregiver and community acceptability of the R21/Matrix-M vaccine alongside existing malaria prevention interventions, according to the communities of participants in the seasonal R21/Matrix-M phase III trial in Mali.

**Methods:**

Qualitative data were collected to assess the acceptability of the R21/Matrix-M vaccine alongside the three R21/Matrix-M or control vaccine priming injections given in the first year of the trial. A total of 33 in-depth interviews (IDIs), 12 focus group discussions (FGDs) and 45 exit interviews at the trial clinics were conducted with caregivers of trial participants, 18 IDIs and 8 FGDs were conducted with community members, 13 IDIs with community health workers and 8 IDIs with trial field staff. Data were coded using the constructs from Sekhon’s theoretical framework on acceptability.

**Results:**

Acceptability of the R21/Matrix-M vaccine was driven mainly by the high burden of malaria in the highly seasonal study area and consequent demand for a malaria vaccine, a perceived high efficacy of the R21/Matrix-M vaccine, and a high level of trust and confidence in the trial and trial team. These perceptions of the acceptability of the R21/Matrix-M vaccine led to a reduced perceived importance of seasonal malaria chemoprevention (SMC) among some caregivers, while others viewed R21/Matrix-M, SMC and insecticide-treated nets as complementary.

**Conclusions:**

The R21/Matrix-M vaccine was acceptable to caregivers and communities of participants in the R21/Matrix-M phase III trial in Mali. Implementation research is needed to evaluate and ensure co-coverage of complementary malaria control interventions, including SMC in seasonal settings, in the face of the scale-up of R21/Matrix-M and other malaria vaccines.

**WHAT IS ALREADY KNOWN ON THIS TOPIC**

- Recently published data from a phase III trial has shown that the R21/Matrix-M malaria vaccine is safe and highly efficacious, and the vaccine has been recommended for use by the WHO.
- The acceptability of the R21/Matrix-M vaccine has not previously been assessed. Previous studies have reported positive community perceptions towards malaria vaccines. However, most of the evidence is from formative research on the perceptions of anticipated malaria vaccines and has not documented the acceptability of actual experiences with malaria vaccines in trial or routine contexts.

**WHAT THIS STUDY ADDS**

- This study investigated the caregiver and community acceptability and factors driving acceptability of the R21/Matrix-M vaccine in a highly seasonal setting during a phase III trial in Mali. The study found a high acceptability of the R21/Matrix-M vaccine in this setting, mainly driven by the burden of malaria and demand for a malaria vaccine, the perceived effectiveness of R21/Matrix-M, and trust in the trial and the trial team.
- In this context where R21/Matrix-M had a high overall acceptability and perceived effectiveness, this study also assessed perceptions of the importance of the use of other malaria interventions alongside R21, such as seasonal malaria chemoprevention (SMC), and found a reduced perceived importance of SMC among some caregivers.
- This study also expands current thinking on how to assess and draw out acceptability of an intervention within a trial context, and the value of assessing acceptability of an intervention alongside efficacy studies, prior to implementation.

**HOW THIS STUDY MIGHT AFFECT RESEARCH, PRACTICE OR POLICY**

- Implementers and policy-makers can use findings on R21/Matrix-M vaccine acceptability and perceptions on the use of R21/Matrix-M alongside SMC and bed nets to help inform the upcoming implementation of R21/Matrix-M and other malaria vaccines. Further implementation research and programme evaluation are needed on the R21/Matrix-M vaccine to maintain the impressive impact achieved with vaccine in trial conditions in the routine setting, including research on its implementation in areas with seasonal malaria transmission and co-coverage with SMC.
- The findings of this study on the understanding of the vaccine within the trial, including the high assumed efficacy of the vaccine, trust in the trial due to previous experiences with research and a low comprehension of randomisation and placebo control, can be used to inform the conduct of future clinical trials.

## Background

 Despite major progress in the fight against malaria over the past decades, progress in recent years has stalled and the burden of malaria remains high. The annual number of malaria cases and deaths has increased globally since 2019, with an estimated 233 million cases and 580 000 deaths in sub-Saharan Africa in 2022.^1^ In 2021, WHO recommended widespread use of the world’s first licensed malaria vaccine, the RTS,S/AS01_E_ vaccine (hereafter referred to as RTS,S), for the prevention of *Plasmodium falciparum* malaria in children.^2^ The recommendation included the potential for countries with seasonal malaria transmission to provide the RTS,S vaccine seasonally.

The R21/Matrix-M vaccine is a novel malaria vaccine. Recently published data from a phase III trial in Mali, Burkina Faso, Kenya and Tanzania showed that the vaccine is safe and highly efficacious.^3^ On the basis of these results, in October 2023, WHO recommended both R21/Matrix-M and RTS,S vaccines for widespread use in the prevention of *P. falciparum* malaria in children.^4^ GAVI has approved funding for a malaria programme and is ready to support roll-out of R21/Matrix-M alongside RTS,S and the manufacturers of R21/Matrix-M have established potential manufacturing capacities of 200 million doses annually.^5^ The R21/Matrix-M vaccine received prequalification by WHO in December 2023 which is a major step towards its deployment at scale. Therefore, it is important to understand the acceptability of the vaccine alongside the current malaria prevention interventions, according to the communities and caregivers of children who will receive the vaccine.

The R21/Matrix-M VAC078 trial is a phase III, double-blind randomised controlled trial.^3^ In 2021, around 1223 children aged 5–36 months of age in the Mali sites were randomised 2:1 to receive either the R21/Matrix-M vaccine or a licensed rabies vaccine (control). A seasonal vaccination schedule was used whereby enrolled children received 3 monthly doses of the trial vaccines in May–July 2021 prior to the malaria transmission season, with an additional booster dose given in June 2022.

The majority of existing evidence on malaria vaccine acceptability is formative research on perceptions of anticipated malaria vaccines and has not documented the acceptability of actual experiences with malaria vaccines in trial or routine contexts.^6^,^11^ Understanding the acceptability of a vaccine within a trial context can help inform successful programme implementation, including the communication strategies needed to promote uptake of the package of the vaccine along with other interventions, while also carefully taking into account the potential impact of the trial context on acceptability of the intervention. Therefore, this study assessed the acceptability of the R21/Matrix-M vaccine, according to the caregivers of participants in the phase III trial and the wider community in Mali. This is the first study to investigate the acceptability of the new R21/Matrix-M malaria vaccine, ahead of the upcoming roll-out of the R21/Matrix-M and RTS,S vaccines in sub-Saharan Africa.

## Methods

**Figure 1:**
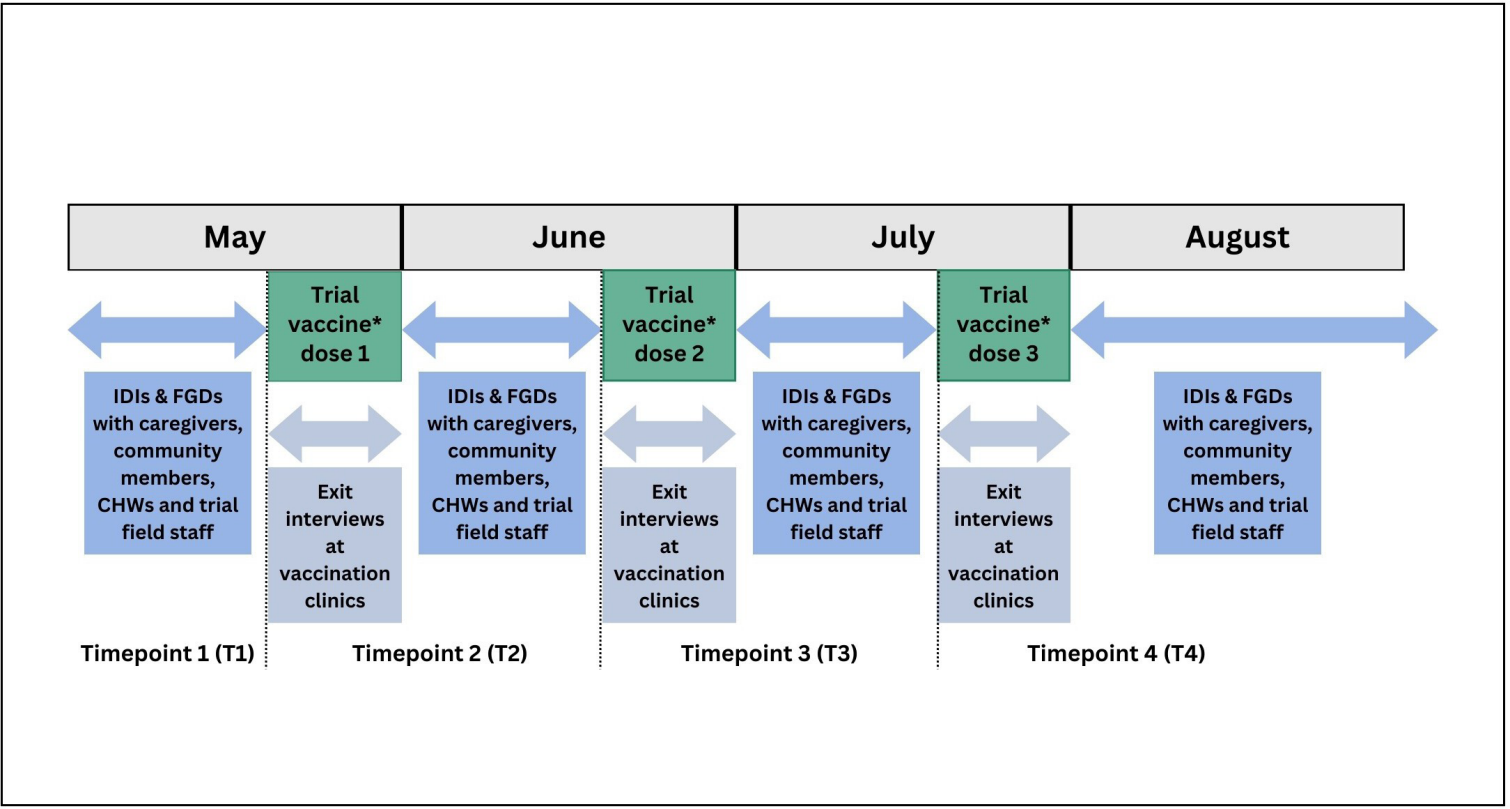
Timing of the qualitative data collection in 2021 with respect to the trial vaccination. *Children in the trial were vaccinated with either R21/Matrix-M or rabies vaccine (placebo) with a 2:1 ratio. SMC was distributed in July and August by the National Malaria Control Programme to trial participants as part of the national routine programme but was not given within the scope of the trial. FGDs, focus group discussions; IDIs, in-depth interviews; SMC, seasonal malaria chemoprevention.

This qualitative study of the acceptability of the R21/Matrix-M vaccine was assessed in the context of the phase III trial, in a seasonal malaria setting. Acceptability was assessed through in-depth interviews (IDIs) and focus group discussions (FGDs) with caregivers of R21/Matrix-M trial participants and community members, exit interviews with caregivers after the trial vaccination clinic visits, and IDIs with community health workers (CHWs) and trial field staff. The IDIs and FGDs were conducted from May to September 2021, prior to (time point 1) and following each of the three trial vaccine priming doses (time points 2–4) (figure 1).

### Study site

The study was conducted in Ouelessebougou and Bougouni districts in Mali, alongside the R21/Matrix-M phase III trial. Ouelessebougou and Bougouni are semirural districts, where agriculture is the main occupation. Malaria is highly seasonal in both districts, with most cases occurring July–November. In the study districts and nationally, malaria is the primary cause of outpatient consultations, hospital admissions and deaths in children under 5 years of age. Insecticide-treated mosquito net (ITN) ownership is high, with around 90% of households owning at least one ITN.^12^ Four monthly cycles of seasonal malaria chemoprevention (SMC) are delivered by the National Malaria Control Programme via door-to-door campaigns during the malaria transmission season from July to October. Nine different childhood immunisations are routinely delivered to children aged 0–23 months by the Essential Programme on Immunisation (EPI) in Mali and vaccine coverage is relatively high, with an estimated 77% of children receiving Penta-3 nationally.^13^ Around one month prior to the start of data collection, the COVID-19 vaccine was introduced in Mali on 31 March 2021, at which point there were 10 042 cases and 385 deaths from COVID-19 reported in Mali.^14 15^

Within the R21/Matrix-M trial, children were followed up through active and passive surveillance and received free 24-hour healthcare at the trial clinics, with transport costs related to the trial or illness covered by the trial. At enrolment, children were given a new ITN. During the trial, SMC was available to the trial participants via the routine NMCP distribution but was not directly given within the scope of the trial. Further details of the trial can be found in Datoo *et al*.^3^

The Malaria Research and Training Centre (MRTC) has conducted research in the districts where the R21/Matrix-M trial was conducted for many years, including the seasonal RTS,S plus SMC trial from 2017 to 2022, a trial of the PfSPZ malaria vaccine in women of childbearing potential and trials of SMC.^16^,^18^ The RTS,S plus SMC and R21/Matrix-M trials were conducted within parts of the same two districts, with some communities participating in both trials. No individual child participated in both trials.

### Respondent selection

The caregivers, community members, CHWs and trial field staff were selected from trial participating sites within the subdistrict health areas in Bougouni (four health areas) and Ouelessebougou (two health areas). These sites were selected to include areas where the RTS,S plus SMC trial^18^ was also being conducted and sites where only the R21/Matrix-M trial was being conducted. Caregivers were selected with assistance from the trial team to include both mothers and male heads of households. Community members were also purposively selected across the six health areas to include variation in gender and literacy. Community members did not have direct contact with the trial but were generally aware of the trial and often knew participants. Between 7 and 10 respondents were selected for each FGD. CHWs were selected from the same health areas as caregivers and community members. CHWs were not directly involved in the trial but assisted the trial team in engaging with the community. Trial field staff were purposively selected to include those with roles that involved the most interaction with the trial participants. At each of the time points, different respondents were selected. Additionally, exit interviews were conducted with trial caregivers as they left the vaccination clinics using convenience sampling after each of the three priming doses, to explore caregivers’ immediate reaction to the vaccination.

### Data collection tools

Discussion guides were used during the IDIs, FGDs and exit interviews for each respondent group to explore themes including caregiver and community acceptability of the R21/Matrix-M vaccine; perceptions of other malaria interventions in the context of the R21/Matrix-M vaccine trial; experiences and perceptions of the trial, including motivators and barriers to participation. The exit interviews included a set of questions on the caregivers’ experience of receiving the vaccine. Additionally, the discussion guides interrogated how contextual factors affected the acceptability of the vaccine, including the context of the R21/Matrix-M vaccine being given through a trial, previous experiences of research including the RTS,S vaccine trial, and contextual perceptions of malaria and malaria interventions, EPI vaccines and COVID-19. The IDIs with CHWs and trial field workers explored their perceptions of caregiver and community acceptability of the R21/Matrix-M vaccine and trial, rather than their own perceptions of the vaccine. The discussion guides were developed using the study investigators’ experience of research surrounding malaria interventions, vaccines and clinical trials and published literature on malaria vaccines and community perceptions of research. Prior to data collection, the discussion guides were piloted and revised.

### Data collection procedures

The interviews and discussions were conducted by four trained MRTC researchers, in Bambara for caregivers, community members and CHWs, and in French for the trial staff. A second trained researcher took field notes of the main points and key observations during the interviews and discussions. During the data collection, field notes were used to review the data and make changes to the discussion guides to include emerging themes for the following time points of data collection. The IDIs, FGDs and exit interviews were conducted either at the respondent’s household, the health facility or a site in the community, depending on the preference and ease of access for the respondent. To prevent the transmission of COVID-19 during data collection, infection prevention and control measures were taken, including social distancing of 1.5 m between respondents and interviewers, hand sanitising with alcohol gel before and after signing consent forms, interviewers and respondents wearing face masks and conducting interviews and FGDs outdoors when possible. All IDIs, FGDs and exit interviews were digitally recorded with the permission of the respondents.

### Data management and analysis

The audio recordings were sent to a professional company for transcription and quality checked by the study team. The interviews in French were transcribed verbatim and the interviews in Bambara were simultaneously transcribed and translated into French. All transcripts were subsequently translated into English and imported into NVivo for coding and analysis. Transcripts were anonymised but the interview number and respondent group were retained to assist the analysis and reporting.

**Figure 2:**
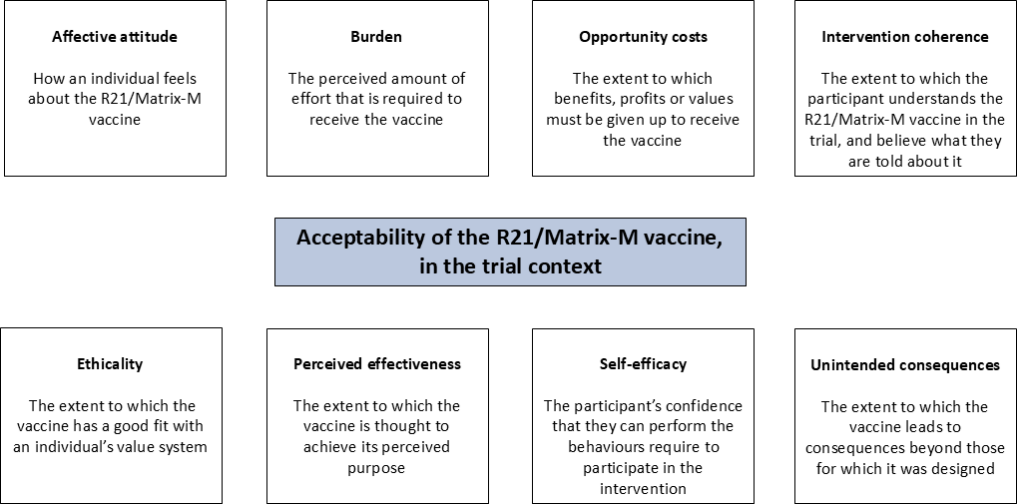
Definitions of the constructs of acceptability,^20 21^ in the context of this study.

The transcripts were coded by one of the study researchers using framework analysis,^19^ with an initial coding framework developed based on the key themes from the interview guides and the adapted version of Sekhon’s framework on acceptability.^20 21^ The adapted framework presents eight constructs of acceptability, affective attitude, burden, opportunity costs, intervention coherence, ethicality, perceived effectiveness, self-efficacy and unintended consequences, which are defined in figure 2. Findings on the perceptions of the overall R21/Matrix-M trial were also coded according to this framework, to understand the trial context and interpret its impact on perceptions of acceptability of the R21/Matrix-M vaccine. These themes were then populated inductively with subthemes as they were identified from the data. The data from each time point were initially coded separately and then synthesised due to only minor differences between the time points.

During analysis, detailed notes were recorded by the coder to inform the interpretation of the results. The coding and the synthesised results were discussed among the researchers at the London School of Hygiene and Tropical Medicine (LSHTM) and MRTC at multiple points during the analysis to help verify the coding and ensure the credibility and confirmability of the findings.

### Patient public involvement

The views and experiences of the caregivers, community members, CHWs and field workers were sought as participants in this study; these groups were not substantially involved in the design or conduct of this study. An author reflexivity statement is provided in online supplemental appendix 1.

The Standards for Reporting Qualitative Research^22^ was used to ensure rigorous reporting of the study (see online supplemental material 1).

## Results

A total of 72 IDIs, 20 FGDs and 45 exit interviews were conducted with caregivers of trial participants, community members, CHWs and trial field workers (total number of respondents=299) (table 1). Summary characteristics of the caregivers can be found in supplement (online supplemental table S1). A summary of the key results on the acceptability of the R21/Matrix-M trial is presented to provide the context within which perceptions of the R21/Matrix-M vaccine acceptability should be interpreted. Key results on the acceptability of the R21/Matrix-M vaccine are presented under each of the four acceptability constructs that were present in the data, alongside illustrative quotes. The results are presented across the respondent groups and time points of data collection as they were generally similar; however, where differences arose, they are discussed and quotes are labelled with the respondent group and time point. Additionally, key themes and quotes on perceptions of the impact of the R21/Matrix-M vaccine on existing malaria prevention interventions are presented.

**Table 1:** Respondents in the IDIs, FGDs and exit interviews

Type of respondent	Number of IDIs, FGDs and exit interviews per time point (T1–4)					Total number of respondents
	T1	T2	T3	T4	Total	
Caregivers of trial participants						
IDIs with female caregivers	5	5	5	6	21	21
IDIs with male caregivers	3	3	3	3	12	12
FGDs with female caregivers	2	2	2	2	8	75
FGDs with male caregivers	1	1	1	1	4	34
Exit interviews with caregivers	–	15	15	15	45	45
Community members						
IDIs with community members	4	5	5	4	18	18
FGDs with community members	2	2	2	2	8	73
Community health workers (IDIs)	4	3	3	3	13	13
Trial field workers (IDIs)	2	2	2	2	8	8
Total number of IDIs, FGDs and exit interviews, and total number of respondents					137	299

FGDsfocus group discussionsIDIsin-depth interviews

### Acceptability of the R21/Matrix-M trial

In general, the caregivers of trial participants and trial communities had positive perceptions of the R21/Matrix-M phase III trial. This was mainly due to the perceived benefits of participating in the trial, including the free, high-quality care provided to the children and the belief that children who participate in trials are healthier (table 2). Respondents reported a strong trust in the trial and the trial team mainly due to the positive experiences and witnessed benefits of previous clinical trials, and the way in which the trial team worked. For most respondents, these benefits and trust outweighed the burden and opportunity costs of attending the trial clinic visits, and any perceived risks of the trial.

**Table 2:** Constructs of acceptability and major themes relating to the phase III R21/Matrix-M vaccine trial

Constructs of acceptability*	Themes
Affective attitude	Free good quality healthcare is given to participants providing financial and emotional relief to caregivers Commodities received (free malaria vaccine, ITN, free healthcare, compensation for time) Trust in trial/trial team built from previous positive experiences with research and the professional, respectful and participatory way in which the team works High demand to participate in the trial and in any future trials Some concerns around blood sampling
Intervention coherence	Poor understanding of placebo and randomisation Good understanding of other principles of research, participation and consent
Perceived effectiveness	Observation that children in trials are generally healthier: no/less frequent/less severe illnesses, when children are ill, receive good treatment and recover quickly
Burden	Long wait times at trial clinic visits, including vaccination visits Not always informed in advance that they need to attend the clinic Sometimes called to clinic and then trial vaccine not available Transport to clinics: provided by trial for rural participants, for others there were difficulties travelling to the clinic, for example, difficulties bringing more than one child and poor roads during rainy season
Opportunity costs	Competing duties at home make prolonged clinic visits challenging Trial visits during busy agriculture period Clinic visits missed due to life events and travel
Unintended consequences	No major risks or concerns associated with trial, having not witnessed anything bad in current or previous trials Trial increased health knowledge and behaviours in community

* No responses associated with the constructs of ethicality and self-efficacy were present in the data.

ITNinsecticide-treated mosquito net

### R21/Matrix-M vaccine and malaria vaccine acceptability

#### Affective attitude

In this study, affective attitude mainly manifested as a perceived need for a malaria vaccine. This demand or need for a vaccine was reported by the majority of respondents across IDIs and FGDs. Perceived burden of malaria to families and communities was a major factor driving this demand, with malaria being seen as the greatest threat to the health of children. Respondents stated that malaria creates major health, emotional and financial burdens for families, whose income and time are spent on treatment and caring for children with malaria.

We have not been able to find effective ways to control malaria so far. When the child suffers, the parents are not at peace. Malaria is our greatest challenge. (male caregiver FGD-2 T1)

There are many benefits [of a malaria vaccine]. When your child is healthy, the income will be better invested. But when the child is sick, all your money goes into treating him or her, and that is often not enough. (community member IDI-3 T3)

Additionally, the seasonality of the burden of malaria was highlighted by respondents, with the malaria transmission season coinciding with the season of intensive farming activities leading to lost opportunity for work.

An additional driver of the demand for a malaria vaccine was that the current methods of preventing malaria, including SMC and ITNs, despite having reduced malaria, are not effective enough and malaria is still the number one cause of childhood illness and deaths. Furthermore, respondents discussed the importance of vaccines in their communities and the belief that vaccines are generally beneficial to the health of children and have been highly effective at ending other diseases, and therefore, could do the same for malaria.

Research has shown that SMC is effective… we are always looking to move forward. If we could have a vaccine against malaria, it will please the members of the community, and if there is a disease that the heads of families are worried about it is malaria. (CHW IDI-2 T1)

We have really seen the benefit of children’s vaccination. We no longer see meningitis and measles; this shows that vaccination is useful. But what we are all waiting for is the vaccine against malaria which is a disease that causes us a lot of suffering. (community member FGD-2 T3)

#### Intervention coherence

While it was widely understood that the trial vaccine protects against malaria, some respondents stated that it also protects against other diseases. This was linked to perceptions of vaccines as a general disease prevention tool that acts against multiple diseases to improve health. Additionally, at times, the symptoms and consequences of malaria, such as fever and anaemia, were viewed as other diseases rather than malaria. During this period of data collection in 2021, respondents also discussed rumours and misinformation circulating in the community about COVID-19 and the COVID-19 vaccine, and the expansion of these fears to other interventions and vaccinations, including the R21/Matrix-M vaccine. Respondents commonly reported hearing fears and rumours that the trial was giving a COVID-19 vaccine instead of a malaria vaccine.

The vaccine [R21/Matrix-M] helps to fight against many diseases including malaria, polio and many other fevers. (caregiver FGD-5 T2)

The fear generated with the appearance of COVID-19 caused people to reject the tablet [SMC]… We have seen neighborhoods refuse EPI vaccines, which have existed since there were diseases throughout the world. There are even women ask us if the R21 vaccine is for COVID-19 (trial field worker IDI-2 T3)

As stated in table 1, the randomisation process and the use of a placebo were not well understood and impacted perceptions of the R21/Matrix-M vaccine. While a minority of caregivers referred to the control vaccine, the majority did not seem to understand this concept and assumed that their child had received the R21/Matrix-M vaccine.

The main objective of the trial and blood sampling are the aspects that people understood well… whether it’s individual or community [consent], it’s extensively explained and understood… the only thing they don't understand is the trial itself because they don't have a high enough intellectual level to understand clinical trials, such as placebo (trial field worker IDI-1 T3)

The malaria vaccine is effective. My child was vaccinated with it and nothing bad happened. (caregiver IDI-2 T2)

#### Perceived effectiveness of the R21/Matrix-M vaccine

Respondents perceived the R21/Matrix-M vaccine to be highly effective in preventing malaria and improving the health of children. Following the second and third priming doses (T3 and T4), caregivers and community members described how they have seen that the children who had been vaccinated in the trial were healthier and no longer had malaria or had malaria less frequently, or less severe malaria than before.

Since my child started participating in this trial, she has not had sickness due to malaria. That’s what I liked… the vaccine trial is going well and our children have been relieved of the malaria disease, they are vaccinated and sleeping under ITNs. (caregiver IDI-6 T3)

At the beginning of the rainy season, there has been a decrease in malaria cases among children… What I know about this vaccine is that my child has received 3 doses and he has had no problem. It protects children against malaria. (caregiver IDI-5 T4)

Prior to the start of the R21/Matrix-M vaccination and during the time of administration of the first dose, there was an assumption that R21/Matrix-M will be effective for multiple reasons, including the general community belief that vaccines are effective and provide the final step in the elimination of diseases. Additionally, in communities where the RTS,S plus SMC trial took place, respondents also described how the RTS,S vaccine was effective against malaria, and the assumption was that R21/Matrix-M will be at least as effective. This was also influenced by the strong level of trust between the communities and trial team. Respondents noted that only benefits had been witnessed from the RTS,S and other previous trials that were seen as successful in reducing malaria, with an assumption that the researchers will continue to bring effective interventions.

According to the communities, they appreciate and believe that R21 also helps to reduce malaria in the community, especially since the RTS,S trial. (trial field worker IDI-2 T4)

The previous study [RTS,S plus SMC trial] reduced malaria in children, which has led to community acceptance; so, people think that the R21 vaccine trial will do even better. (CHW IDI-2 T4)

#### Unintended consequences

Caregivers commonly reported that their child had experienced side effects following vaccination in the trial, such as fever. While caregivers disliked the side effects and the burden of having to take the child back to the health centre for care for the side effects, they did not cause major concerns for most caregivers and so did not impact substantially on the acceptability of the vaccine. Caregivers mostly described the side effects as mild, which went away when given medicine. Additionally, caregivers reported that they were used to these mild side effects from routine EPI vaccines, understood that they were not dangerous and knew how to treat the side effects. Caregivers also discussed how the side effects were not concerning as they were informed about them before vaccination by the trial staff and the children were looked after by the trial if they had side effects.

Respondent (R)1: After the administration of the vaccine, the doctors come back to see if the child has had any problems. If there were, they would give them medicine… R10: There is no problem with the R21 vaccine. After the administration of the vaccine, the child will have a fever, but everything will be fine afterwards. (caregivers FGD-3 T4)

The children that get vaccinated experience some side effects like inflammation or digestive problems, but the mothers already know these side effects from the community EPI vaccination, these effects are comparable to the effects of the R21 vaccine, which means that the mothers are not concerned about these effects. (Trial field worker IDI-2 T3)

However, while the caregivers who took the child for vaccination were counselled about the possible side effects and what to do if they occurred and so did not have concerns, there were some concerns surrounding the side effects from others who had not received these communications; as most caregivers taking the children to the trial visits were mothers, some male caregivers said that they were not told about the side effects and did not understand why a vaccine would cause these illnesses. Some community members also had concerns about the possibility of more serious vaccine side effects and reported some caregivers being frightened by what they had heard about the side effects and wanting to leave the trial.

R5: we struggle to understand why children have fever after the vaccination, they should do everything to avoid fever. R6: The purpose of the vaccine is to prevent disease, not to cause it… R1: You should have told us about the side effects that it causes. So, the problem is related to communication. (male caregivers FGD-4 T4)

Some people are in this trial and they want to leave because the vaccine makes their children sick. I am trying to make them aware of the need to stay. (community member FGD-5 T4)

### Impact of the R21/Matrix-M vaccine on perceptions of existing malaria prevention interventions

The R21/Matrix-M vaccine will be rolled out alongside existing malaria prevention interventions, and as such it was important to understand respondents’ perceptions of SMC and ITNs alongside the R21/Matrix-M vaccine.

#### Seasonal malaria chemoprevention

Caregivers of trial participants discussed their perceptions of the importance of SMC after their child received the trial vaccine, which as discussed above was widely understood to be the R21/Matrix-M vaccine, but with limited understanding of the randomisation and control vaccination. These perceptions were influenced by three main factors, the acceptability of the R21/Matrix-M vaccine, the acceptability of SMC in the study area and the trial context, including understanding of the trial and the communications they received from the trial staff (table 3).

**Table 3:** Impact of receiving the trial vaccine on caregivers’ perceptions of the importance of SMC

Caregivers’ perceived importance of SMC after receiving trial vaccine			Illustrative quotes
Important for child to take SMC	Acceptability of R21/Matrix-M vaccine	R21/Matrix-M and SMC complement each other—together they enhance protection against malaria	'malaria is an endemic disease, the vaccine can prevent it to some extent, and SMC is a drug that prevents malaria as well, so they are complementary' (caregiver EI-8 T2)'The vaccine works on the child’s body, but the SMC helps fight the germs of diseases inside the child’s belly. It will make the child feel safe. It is very important.' (caregiver FGD-1 T3)
	Acceptability of SMC	SMC is effective at preventing malaria and improving the health of children	'R4: The SMC has considerably reduced malaria, especially during the rainy season, when children get sick a lot. R2: It is important to take the SMC because when you give it to the child you could quietly go to the field without worrying about the child’s health. We look forward to always having it. That’s why the SMC is important.' (caregiver FGD-4 T1)'during the rainy season there are many diseases, if you refuse to give the SMC to your child because he has received the malaria vaccine, SMC fights against diseases during this period too. If you are given these tablets, you must give them to your child. You will see that he will make it through this period without getting sick, and the SMC has many advantages' (caregiver IDI-6 T1)
		SMC is effective against other illnesses as well as malaria	
	Understanding of and communications from trial	Caregivers told by trial workers to give their child SMC	'(SMC) is important, because the vaccine is a trial, so a trial doesn't prevent a disease… In my opinion, one does not stop the other, each one (SMC and R21) has its importance… because it is difficult to get rid of malaria since the vaccine is in the trial phase.' (caregiver IDI-5 T2)
		Because the vaccine is in the trial phase, they should still take SMC	
Not important for child to take SMC	Acceptability of R21/Matrix-M vaccine	R21/Matrix-M and SMC do the same thing	'If the child receives the vaccine it is no longer important that he/she takes the SMC, because both products play the same role.’ (caregiver EI-3 T4)'We believe that this vaccination (R21/Matrix-M) will be as successful and beneficial as previous vaccinations. Vaccination is more effective than tablets (SMC)' (male caregiver FGD-2 T1)
		R21/Matrix-M is more effective than SMC and will replace SMC	
	Acceptability of SMC	Do not like SMC due to side effects—now they have received the vaccine they will not give them SMC anymore	'People like the malaria vaccine, but there have mixed feelings about SMC. Some people say it protects children from malaria, but others say it makes children sick.' (CHW IDI-3 T3)'Actually, I don't give SMC to my child. When I give it to her, she gets a fever, that' s why I refuse to give it to her.' (caregiver EI-13 T2)'The truth is, we don't give that (SMC) to our kids. Their father is against it. He said he does not accept that his child is given street drugs. He said he does not want his children to take the medication that the health workers give door-to-door.' (caregiver EI-12 T4)
		Do not like SMC due to side effects—do not give to child	
		Refuse SMC due to perceptions and suspicions of them being low quality as they are free medicines delivered to your home, and not in the health facility	
	Understanding of and communications from trial	When enrolled in a trial, your children should only take drugs given by the trial team	'I would not be able to give my child this medicine (SMC) unless the trial staff gave me permission. They are the ones who know if it is important for the child or not.' (caregiver EI-6 T4)'It’s not necessary for her to take that (SMC) anymore. So that the impact of the vaccine can be better assessed' (caregiver EI-15 T4)
		Because it is research children should not take SMC so the researchers can see how well the vaccine is working	

SMCseasonal malaria chemoprevention

Generally, there was a high level of acceptability of SMC, mainly due to the perceived protective effect of the intervention on malaria in children observed over multiple years of implementation. Many caregivers still perceived it to be important for their child to receive SMC following trial vaccination, with the belief that R21/Matrix-M and SMC are complementary and together, enhance protection. However, there were also existing negative perceptions of SMC, primarily due to its side effects, including vomiting, fever and diarrhoea. Some caregivers stated that because of this they have refused SMC or thrown it away after receiving it from the health workers. Others said they had given SMC but would now stop as their child had been vaccinated. It was commonly expressed by other caregivers that they do not believe SMC is needed once the child received the vaccine, as the interventions act to prevent malaria in a similar way and vaccines are more effective. A smaller number of caregivers were influenced by factors surrounding the trial context, including that they were told by some trial workers, who they trust, to take SMC delivered by the national programme, while other caregivers said that they should only let their child take drugs they receive from the trial field workers. Illustrative quotes to support these results are provided in table 3.

#### Insecticide-treated nets and care-seeking behaviour

Unlike the situation with SMC, almost all caregivers felt that it was important for their children to sleep under an ITN after vaccination. This was because ITNs were felt to be well accepted and established in the communities, preventing other diseases beyond malaria and against the biting nuisance of insects. Additionally, ITNs were commonly seen to provide a different type of protection to R21/Matrix-M vaccination and SMC, protecting against the cause of the disease, so were seen to be complementary, and even sometimes necessary for the vaccine to work. However, two caregivers stated that ITNs were no longer needed as the R21/Matrix-M vaccine would sufficiently protect against malaria. Some caregivers also said they would use ITNs after vaccination because they had been given one by the trial team and told to use it.

If the child is vaccinated against malaria and sleeps under the net, both help fight malaria. If the child is vaccinated and does not sleep under the net and is bitten by mosquitoes, they will eventually get malaria. (caregiver FGD-2 T2)

Mosquitoes are the cause of malaria. If the child doesn't sleep under a net, after the vaccination, it will be useless; they should continue to sleep under an impregnated mosquito net. (caregiver IDI-6 T2)

Caregivers were also asked about their perceptions of the importance of seeking care if their child had fever or other symptoms. However, this was strongly influenced by the trial context as trial participants were given free high-quality care by the trial staff, transport to the clinic if needed and strongly encouraged to bring their child to the trial clinic if they were unwell. Nevertheless, receiving the vaccine did impact on whether caregivers thought that fever in their child was likely to be malaria, as many caregivers mentioned that it was still important to seek care after their child received the vaccine, as they could have other illnesses besides malaria.

It is important to still go to the health centre if your child is sick. After the malaria vaccine, the child may still have another disease besides malaria. (caregiver IDI-6 T3)

## Discussion

This study found that the R21/Matrix-M malaria vaccine was very acceptable to the caregivers of trial participants and trial participants’ communities. This was driven mainly by the recognised burden of malaria, the need for a malaria vaccine, the perceived high effectiveness of the vaccine and the trust and confidence in the trial and the trial team. The introduction of a malaria vaccine was viewed as the final tool needed to prevent malaria as a major public health problem, as observed following the introduction of other childhood vaccines in the communities, with diseases such as measles.

Previous studies have reported generally positive community perceptions towards potential malaria vaccines.^6^,^11^ However, most of this evidence is formative research on perceptions of anticipated malaria vaccines and has not documented acceptability of actual experiences with malaria vaccines in trial or routine contexts. This is the first study to investigate the acceptability of the new R21/Matrix-M malaria vaccine, ahead of the upcoming roll-out of the R21/Matrix-M and RTS,S vaccines in sub-Saharan Africa.

In the phase III trial in Mali, the R21/Matrix-M vaccine was given according to a seasonal rather than an age-based schedule. Given the highly visible seasonality of malaria in the study site, there was a positive attitude among the communities towards seasonal malaria vaccination. This overall affective attitude towards a malaria vaccine contributed to the high perceived effectiveness of the R21/Matrix-M vaccine observed in the study, which was both assumed and witnessed by respondents and was present even while the first vaccine doses were being administered. This perceived effectiveness was due to the belief that childhood vaccines in general are effective and the demand for a malaria vaccine, and was influenced by the high efficacy of RTS,S reported in the trial site.^18^ Following the second dose of the vaccine in June in the R21/Matrix-M trial, respondents also reported a visible effect of the vaccine in reducing malaria and general illness. Although a visible effect within such a short time is plausible in this seasonal malaria context, it is likely that this was due to a combined effect of the R21/Matrix-M vaccine (given to two-thirds of the trial children and which provided 82% protection against clinical malaria in the Mali site over a 12-month follow-up period^3^), the distribution of ITNs, improved knowledge and behaviour around malaria prevention, and also, importantly, early healthcare seeking with high-quality care provided.

Acceptability of the vaccine was influenced by the trust and confidence the respondents had in the trial and trial team, and the interventions that they test. Previous studies have found that prior experiences with research can both positively and negatively impact perceptions of studies.^23^ In the current case, trust in the MRTC trial team had been built up over several years from the perceived success of previous trials in reducing malaria and improving the health of communities and the transparent, respectful and participatory approach of the team. Similarly, outside of the trial setting, trust in the health workers delivering vaccines and the wider health system is a well-reported factor affecting the uptake of other childhood vaccines.^24^

Understanding of the concept of randomisation and a placebo control was low among caregivers of trial participants as noted in other settings; a systematic review of studies conducted in Africa found poor comprehension of key concepts of informed consent, including randomisation and placebo.^25^ A study conducted alongside a phase III trial of the RTS,S vaccine in Kenya, 1 year after consent had been obtained, showed that only 15% of caregivers reported that not all study children had received the malaria vaccine, and only 15% reported that children who had received the trial vaccine were still able to get malaria.^26^ Approaches that have been suggested to improve the consent process and understanding of research include using digital technologies to increase engagement and comprehension during consenting, using tools to assess areas of miscomprehension, greater flexibility in ensuring suitability for different sociocultural contexts, as well as supporting informed consent with community engagement.^27^,^30^

Caregivers generally believed that their children were receiving an efficacious malaria vaccine in the trial, and this influenced their perceptions of the importance of other malaria prevention interventions, in particular SMC. While some caregivers perceived SMC as complementary to R21/Matrix-M, others felt that since their child had received the vaccine, receiving SMC following the third dose of the vaccine was no longer important. Their reasoning for this was that the two interventions were believed to act in a similar way, with vaccines being more effective and SMC sometimes causing side effects. These findings have important implications for the upcoming roll-out of malaria vaccines where malaria vaccines are introduced within the package of existing malaria prevention interventions, including SMC. The seasonal RTS,S plus SMC trial in Mali and Burkina Faso found that the combination of seasonal RTS,S vaccine and SMC provided around 60%–70% greater protection against uncomplicated and severe malaria and deaths from malaria, when compared with SMC or RTS,S alone.^18^ The RTS,S vaccine pilot implementation programme, which introduced RTS,S only in areas without SMC, did not find any impact on the use of ITNs or health-seeking behaviour following the introduction of the vaccine, which included careful messaging about the partial efficacy of the vaccine and the importance of continued use of current malaria prevention interventions.^2^ Additionally, the pilot found that the introduction of the malaria vaccine expanded access to at least one malaria prevention tool, and this could be the case for SMC if it continues to be delivered in areas with highly seasonal malaria together with a malaria vaccine. Caregivers in the R21/Matrix-M trial were widely accepting of the combination of R21/Matrix-M and ITNs, but with more divergence of opinion on the combination of R21/Matrix-M and SMC as these were perceived to act in a more similar manner. Additionally, there were background issues with the acceptability of SMC, due to the side effects, as reported in other studies.^31^ These side effects, alongside positive attitudes towards vaccines, led caregivers to prefer the R21/Matrix-M vaccine as an alternative to SMC. As R21/Matrix-M and RTS,S are rolled out beyond the pilot areas, including in areas with SMC and perennial malaria chemoprevention, programmes should carefully consider and assess the effect that malaria vaccines may have on the uptake of SMC and other malaria interventions. Integration of implementation activities, such as integrated communications and careful messaging around the partial protectivity of the vaccine and the need for the combined interventions, may support the uptake of the combined interventions. Additionally, remaining barriers to SMC acceptability and administration should be further investigated in these contexts to achieve high coverage of both interventions.

Acceptability of the R21/Matrix-M vaccine was examined in the context of a phase III trial in an area with highly seasonal malaria transmission. Therefore, the results must be interpreted within this context, including the perceived benefits of participation from the current and previous malaria trials such as the free high-quality care, and the trust and confidence in the trial and trial team. Through presenting the perceptions of acceptability of the R21/Matrix-M vaccine, alongside a summary of the overall trial acceptability, this study provides an example of how to interpret the acceptability of an intervention in a trial context. Limited research has been conducted on assessing vaccine acceptability in the context of a malaria vaccine efficacy trial.^32^ This study provides useful findings to inform implementation through assessing acceptability alongside an efficacy study. However, implementation research is needed on how best to deliver the R21/Matrix-M or RTS,S vaccine, including how to deliver it in areas with seasonal malaria transmission.^33 34^ Previous studies, including one undertaken as component of the RTS,S pilot programme, have shown that high demand and acceptability for a vaccine do not always translate to uptake of the vaccine in the face of health system barriers, and implementation research and programme evaluation are needed to identify context-appropriate strategies to achieve high uptake.^35^

This study used an adapted version of Sekhon’s acceptability framework to present the findings on the acceptability of the R21/Matrix-M vaccine, and summary of the key themes related to the acceptability of the overall trial. Data on four of the constructs were found in the transcripts on the acceptability of the R21/Matrix-M vaccine, affective attitude, which mainly presented as demand or perceived need, perceived effectiveness, intervention coherence and unintended consequences. The constructs burden and opportunity costs, that are intrinsically more related to the delivery of an intervention like a vaccine than the intervention itself, were not found in the data for the R21/Matrix-M vaccine, as the burden and opportunity costs of receiving the trial vaccine were embedded within the overall burden of trial participation, including non-vaccination contacts and procedures. However, these barriers to acceptability and uptake of vaccines, including long wait times at clinics, issues with transport to clinics and competing work and life events, especially during busy farming periods, are also commonly reported barriers to the acceptability and uptake of vaccines in the routine setting.^24^

### Limitations

The findings of this study are limited by the fact that data were only collected just prior to, during and immediately following the three priming doses of the trial vaccine, and the first 2–3 months of the malaria transmission season, during two of which (July and August) SMC was also administered. Following this, the trial gave booster doses of R21/Matrix-M prior to the transmission season in 2022 and 2023. It is possible that the acceptability of the vaccine, including its perceived effectiveness and perceptions of SMC administration alongside the vaccine, may have changed over time through the malaria transmission seasons, and the administration of additional doses. Social desirability bias may have also influenced responses as the data collectors were from the same research institute as the trial team, and the trial field workers interviewed were members of the trial team.

## Conclusions

Acceptability of the R21/Matrix-M vaccine was high among caregivers and communities participating in the seasonal R21/Matrix-M phase III trial in Mali. This acceptability was driven mainly by the high burden of malaria and demand for a malaria vaccine, the perceived high efficacy of the R21/Matrix-M vaccine, and the trust and confidence in the trial and trial team. This high acceptability of the R21/Matrix-M vaccine resulted in a reduction in the perceived importance of SMC among some trial caregivers, while other caregivers viewed R21/Matrix-M, SMC and ITNs as complementary. While this study was conducted in a trial setting, it includes important findings that can inform both the conduct of future clinical trials, and the routine implementation of R21/Matrix-M and other malaria vaccines. Further research and programme evaluation are needed on the upcoming implementation of the R21/Matrix-M vaccine to maintain the impressive impact achieved with vaccine in trial conditions in routine settings. Additionally, implementation research is needed to investigate how to integrate malaria vaccination into existing programmes already delivering other effective malaria control interventions and ensure co-coverage of complementary malaria control interventions in the scale up of R21/Matrix-M.

## supplementary material

10.1136/bmjgh-2024-015524 online supplemental appendix 1

10.1136/bmjgh-2024-015524 online supplemental material 1

## Data Availability

Data are available on reasonable request.
