# Supplementary material for: Acceptability of the R21/Matrix-M malaria vaccine alongside existing malaria interventions in the trial context
Source: BMJ Glob Health. 2025 Feb 3;10(2):e015524. doi: 10.1136/bmjgh-2024-015524 (PMC11795384; doi:10.1136/bmjgh-2024-015524)
Supplement: online supplemental appendix 1 [file bmjgh-10-2-s001.pdf]

SUPPLEMENTAL MATERIAL

1. Author reflexivity statement
2. Table S1: Socio-demographic information of study participants, by participant group

Author Reflexivity Statement

1. How does this study address local research and policy priorities?

Malaria is the leading cause of outpatient consultations, hospitalisations and deaths in children in Mali. This burden remains despite the wide-scale implementation of effective preventive interventions. The goal set forth by the National Malaria Control Programme and Mali's Ministry of Health is to eradicate malaria in Mali by 2030; new approaches to malaria prevention will be needed to meet this goal. Mali was one of the four countries in which the clinical trial demonstrating the efficacy and safety of the R21/Matrix-M malaria vaccine was conducted. The vaccine has now been recommended by WHO and the Mali Ministry of Health (MoH), and other West African MoH, are highly interested in the potential implementation of R21/Matrix-M, and have applied to GAVI for supply of malaria vaccines. This study provides findings on the acceptability of the R21/Matrix-M vaccine alongside the current malaria prevention interventions, according to the communities and caregivers of children who will receive the vaccine in Mali. This information can be used by decision makers and implementers in Mali to inform the potential upcoming implementation of R21/Matrix-M and other malaria vaccines.

2. How were local researchers involved in study design?

The conceptualisation of the study and development of the protocol and study materials were done jointly by the authors in Mali (HD, ST, AD, IS) and in the U.K. (JG, JW, BG, DC, MD, AVSH). All these researchers made substantial and important contributions to the study design.

3. How has funding been used to support the local research team?

This project has supported the development of an experienced social science team at the Malaria Research and Training Centre (MRTC) in Mali. Additionally, this project supported training for one Master's student (ST) at Ouagadougou Training and Research Institute Interdisciplinary in Science Health and Education (IFRISSE) and two medical students from the University of Sciences, Techniques and Technologies of Bamako in qualitative research and gave them practical experience of qualitative data collection.

4. How are research staff who conducted data collection acknowledged?

The members of the research team who significantly contributed to the data collection are authors ST, HD, FK, DI, MS and ABD as stated in the contributions section. Other researchers who helped to collect the data did not significantly contribute to shaping how the data were collected, or how the study findings were interpreted, are thanked in the acknowledgements section.

5. Do all members of the research partnership have access to study data?

All members of the partnership have access to the study data through JG, ST, HD and JW.

6. How was data used to develop analytical skills within the partnership?

This was not specifically addressed during the study.

7. How have research partners collaborated in interpreting study data?

Interpretation of the data was a collective effort from the authors. In-person, and virtual meetings were held during data collection and analysis between HD, ST, JG, JW and FK to discuss the interpretation of the study findings. The findings were presented to the other authors and the wider R21/Matrix-M Phase III Trial Group during the study to gain their input.

8. How were research partners supported to develop writing skills?

JG and HD wrote the first draft of the manuscript and was guided and supported in this by JW and other senior authors. All authors reviewed the manuscript and made substantial contributions to it.

9. How will research products be shared to address local needs?

The preliminary results of this study were disseminated to the MRTC team in Mali. The final results of the study presented in the manuscript will be disseminated to key Ministry of Health staff, especially the malaria and immunisation stakeholders at national, regional and district in Mali, and more widely in West Africa.

10. **How is the leadership, contribution and ownership of this work by LMIC researchers recognised within the authorship?**

Eight out of 14 of the authors are from Mali, including the co-first author.

11. **How have early career researchers across the partnership been included within the authorship team?**

We have included early career researchers from both partner organisations (JG, ST, FK) within the authorship team. The early career researchers contributed to all stages of the study, from study conception to analysis and paper-writing.

12. How has gender balance been addressed within the authorship?

Five of the authors are female (JG, FK, MD, JW, HD) and nine are male (ST, DI, ,MS, ABD, IS, DC, AD, AVSH, BG).

13. How has the project contributed to training of LMIC researchers?

JW, an experienced social science researcher, mentored the team throughout the study. HD and ST provided training on qualitative techniques, supervision and support to FK and the other data collectors. ST was supported by HD, AD, JW and JG to gain experience as a social science researcher and to advance in this field.

14. How has the project contributed to improvements in local infrastructure?

This project has not directly contributed to improvements in local infrastructure.

15. What safeguarding procedures were used to protect local study participants and researchers?

All study processes, including data collection, storage, analysis, and sharing, followed the principles and guidelines laid down by the ethics committee of the Faculty of Medicine, Pharmacy and Dentistry, University of Bamako, Mali, and the ethics committee of the London School of Hygiene and Tropical Medicine, UK.

Table S1: Socio-demographic information of study participants, by participant group

	**Caregivers of trial participants**	**Community members**	**Community health workers**
District, N (%)			
Bougouni	95 (51)	49 (54)	7 (54)
Ouelessebougou	92 (49)	42 (46)	6 (46)
Sex, N (%)			
Female	141 (75)	60 (66)	4 (31)
Male	46 (25)	31 (34)	9 (69)
Relationship to trial child, N (%)			
Mother	137 (73)		
Father	38 (20)		
Grandparent	8 (5)		
Aunt/uncle	4 (2)		
Highest level of education started, N (%)			
No education	84 (45)	41 (45)	1 (8)
Arabic school	9 (5)	2 (2)	1 (8)
Primary school	62 (33)	26 (29)	5 (38)
Secondary school	13 (7)	7 (8)	6 (46)
Tertiary/higher	2 (1)	1 (1)	-
Reading and writing in local language classes	12 (7)	10 (11)	-
Other (Islamic learning, informal learning at home)	4 (2)	4 (4)	-
Profession, N (%)			
Housewife	106 (57)	43 (47)	1 (8)
Farmer	29 (16)	14 (15)	6 (45)
Vendor	25 (13)	16 (18)	-
Skilled craft trader	14 (8)	10 (11)	1 (8)
Salaried worker	3 (2)	2 (2)	1 (8)
Medically trained professional	-	-	4 (31)
Other*	8 (4)	6 (7)	-

*student (7), retired (5), real estate agent (1), traditional healer (1)
