# Supplementary material for: Acceptability of the R21/Matrix-M malaria vaccine alongside existing malaria interventions in the trial context
Source: BMJ Glob Health. 2025 Feb 3;10(2):e015524. doi: 10.1136/bmjgh-2024-015524 (PMC11795384; doi:10.1136/bmjgh-2024-015524)
Supplement: online supplemental material 1 [file bmjgh-10-2-s002.pdf]

The Standards for Reporting Qualitative Research (SRQR) checklist

**Title and abstract**	
1.Title	Includes a concise description of the topic of the study and reflects the type of study p1. While the type of study is reflected in the title that is, an acceptability study, it is also clearly stated in the abstract and early in the methods section that this is a qualitative study.
2. Abstract	Abstract includes the background and purpose (aim), methods, results and conclusions p2
**Introduction**	
3. Problem formulation	Significance of the problem/phenomenon studied p3; relevant theory (analytical framework) p5-6; problem statement p3
4. Purpose or research question	Purpose (aim) p3
**Methods**	
5. Qualitative approach and research paradigm	Qualitative approach and theory p4-6
6. Researcher characteristics and reflexivity	Researcher characteristics and institutional background p1 and p5
7. Context	Study setting and salient contextual factors p4
8. Sampling strategy	Selection of study sites and participants p4-5
9. Ethical issues pertaining to human subjects	Ethics approval from two ethics committees and informed consent p17
10. Data collection methods	Data collection procedures p4-5
11. Data collection instruments and technologies	Discussion guide details p5
12. Units of study	Type of participants and number of participants p5-7
13. Data processing	Data management prior to analysis p5
14. Data analysis	Description of coding and analysis process and verification by wider team p5-6
15. Techniques to enhance trustworthiness	Analysis conducted and reviewed by multiple authors to help validate the findings and interpretation p6
**Results/Findings**	
16. Synthesis and interpretation	Main findings p6-11
17. Links to empirical data	Illustrative quotes from participants are presented throughout the results.
**Discussion**	
18. Integration with prior work, implications, transferability, and contributions to the field	Summary of main findings and how they connect to and elaborate on earlier scholarship p11-13. Discussion of generalisability p13. Identification of unique contribution to scholarship p3, p11 and p12-13.
19. Limitations	Limitations p13
**Other**	
20. Conflicts of interest	None to declare p14
21. Funding	University of Oxford (which received funding in turn from the Serum Institute of India). p14
